# Direct Determination of Glyphosate and Its Metabolites in Foods of Animal Origin by Liquid Chromatography–Tandem Mass Spectrometry

**DOI:** 10.3390/foods13152451

**Published:** 2024-08-02

**Authors:** Marija Denžić Lugomer, Nina Bilandžić, Damir Pavliček, Tiana Novosel

**Affiliations:** 1 Laboratory for Analytical Chemistry and Residues, Veterinary Department Križevci, Croatian Veterinary Institute, 48260 Križevci, Croatia; 2 Laboratory for Residues, Department of Veterinary Public Health, Croatian Veterinary Institute, 10000 Zagreb, Croatia

**Keywords:** glyphosate, AMPA, N-acetyl-glyphosate, N-acetyl-AMPA, LC-MS/MS, food of animal origin

## Abstract

Glyphosate is the most used herbicide in agriculture. Its major metabolite is AMPA (aminomethylphosphonic acid), but N-acetyl-AMPA and N-acetylglyphosate are also metabolites of interest. For risk assessment, a general residue definition was proposed as the sum of glyphosate, AMPA, N-acetyl-glyphosate and N-acetyl-AMPA, expressed as glyphosate. A confirmatory method for glyphosate in fat, liver and kidneys, as well as a confirmatory method for AMPA and N-acetyl-glyphosate in all matrices, are still missing. In this paper, we present a method for the quantitative determination of glyphosate residues and its metabolites AMPA, N-acetyl-AMPA and N-acetyl-glyphosate by liquid chromatography–mass spectrometry (LC-MS/MS) in adipose tissue, liver, eggs, milk and honey without derivatization. Different chromatographic columns were tested, with the Hypercarb column providing the best results. The analytes were eluted with mobile phases of acidified water with 1.2% formic acid and 0.5% formic acid in acetonitrile. Sample purification procedures were also optimized by varying the solvent extraction mixtures (water, methanol and mixture ψ (methanol, water) = 1:1, each with the addition of 1% formic acid (*v*/*v*)), using different sorbents in solid phase extraction (SPE) (polymeric cationic (PCX) and anionic (PAX)) and using dispersive solid phase extraction (dSPE) (C18 and PSA) by modifying the extraction procedures. Finally, the analytes were extracted from the samples with 1% formic acid in water (*v*/*v*). Milk and adipose tissue were purified by the addition of dichloromethane, while liver and egg samples were purified by SPE with a mixed cation exchange sorbent and ultrafiltration with cut-off filters. The proposed analytical procedures were validated according to SANTE/11312/2021 guidelines: linearity, limits of quantification, precision and accuracy were determined for all matrices. The limits of quantification (LOQs) ranged from 0.025 to 0.2 mg kg^−1^. Precision, expressed as relative standard deviation, was <20%, while accuracy, expressed as analytical recovery, ranged from 70% to 120%. During method validation, the measurement uncertainty was estimated to be <50% for all analytes. Good validation parameters according to the SANTE document were achieved for all analytes. Therefore, the method can be considered reliable and sensitive enough for routine monitoring of polar pesticides. The application of the accredited method in routine analysis will provide data that are useful for the re-evaluation of risk assessment studies in foods of animal origin.

## 1. Introduction

**Figure 1:**
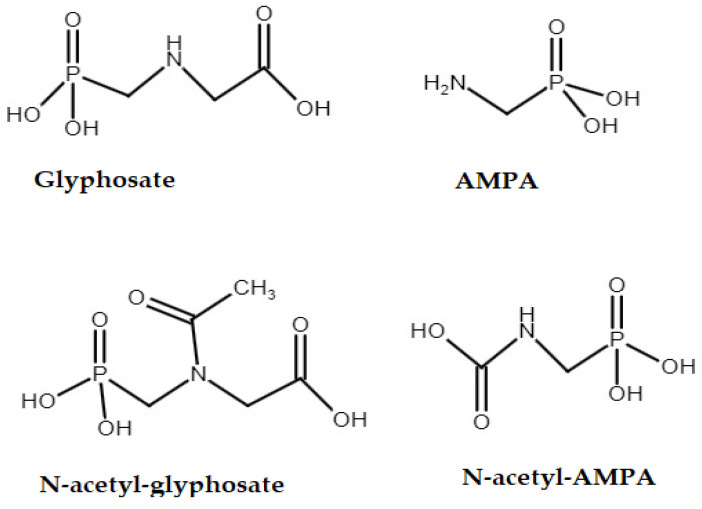
Chemical structure of glyphosate and its metabolites.

Glyphosate (N-(phosphonomethyl)glycine) is a non-selective herbicide that is widely used in agriculture and industry and acts as an inhibitor of the enzyme 5-enolpyruvylshikimate-3-phosphate synthase in the shikimate pathway. Its great success is due to its high efficiency in eliminating weeds, making glyphosate-based herbicides the most widely used pesticide in the world. As glyphosate is approved for use on various crops that can be fed to animals, there is a possibility that it forms residues in foods of animal origin. Aminomethylphosphonic acid (AMPA) is the main metabolite of glyphosate and is therefore often analysed together with glyphosate [1]. Other metabolites of glyphosate are N-acetylglyphosate and N-acetyl-AMPA, which are formed in glyphosate-tolerant plants, i.e., in genetically modified plants that contain the modification glycine-N-phenylacetyltransferase (GAT) (Figure 1). GAT inactivates glyphosate by converting it into N-acetyl-glyphosate, so that N-acetyl-glyphosate is the main metabolite in these plant products. It is assumed that N-acetyl glyphosate is further degraded to N-acetyl AMPA and AMPA.

In Europe, maximum residue levels for pesticides apply to all foodstuffs intended for human consumption in order to protect human health. The European Food Safety Authority (EFSA) has set the limit of analytical quantification (LOQ), which could correspond to the MRL for glyphosate in animal raw materials such as animal tissue, at 0.05 mg kg^−1^ [2,3]. The EFSA also proposed to consider the following residue definition: the sum of glyphosate, AMPA and N-acetyl-glyphosate expressed as glyphosate for monitoring, as well as the sum of glyphosate, AMPA, N-acetyl-glyphosate and N-acetyl-AMPA expressed as glyphosate for risk assessment. The sum of glyphosate, AMPA and N-acetyl glyphosate, expressed as glyphosate, can be enforced at the combined LOQ of 0.2 mg kg^−1^ in all matrices (Table 1). However, the lack of information on the presence of glyphosate and its metabolites in products of animal origin means that the MRLs still have to be considered as provisional. To close this gap, the EFSA emphasises the need for confirmatory methods for glyphosate, AMPA and N-acetyl-glyphosate in fat, liver and kidneys and a confirmatory method for AMPA and N-acetyl-glyphosate in all matrices. To ensure food safety and assess consumer exposure to pesticide residues in foods of plant and animal origin, multi-annual monitoring programmes for pesticide residues are carried out by all member states [4].

Due to the high polarity of glyphosate and AMPA, chromatographic separation is a difficult task, and it is even more difficult to combine highly polar pesticides with less polar pesticides such as N-acetyl-AMPA and N-acetyl-glyphosate [5]. For these reasons, glyphosate cannot be detected with the multi-residue methods used in pesticide analysis; it is analysed with so-called single residue methods (SRMs).

The residue determination of glyphosate and AMPA is extremely problematic due to their amphoteric nature, their low mass and the absence of chemical groups that could facilitate their cleavage. All these factors complicate their analysis by HPLC with UV or fluorescence detectors and GC MS without derivatization. Most methods developed to date use pre- or post-column derivatization to form fluorescent derivatives and/or reduce the polar character of the analyte, which facilitates its chromatographic retention [6,7]. Derivatization procedures are usually time consuming and require hazardous chemicals, making the methods unsustainable. Typical C18 or C8 inverted phase silicate columns have a problem with retention of such polar compounds, resulting in unresolved co-eluting peaks with polar analytes eluting into the dead volume of the column. Therefore, special chromatography columns must be used for non-derivatized analytes such as HILIC (hydrophilic interaction liquid chromatography), porous carbon graphite such as Hypercarb [5,8,9,10,11], anion exchange columns and mixed-mode columns that combine reversed-phase properties and weak anion and/or cation exchange in one [11,12,13,14,15]. When using the mentioned columns, a problem often occurs after several sample injections: the peaks for glyphosate become broader and excessively elongated, indicating a stronger retention of the analyte. The Hypercarb column consists of 100% porous graphitized carbon and is used for the analysis of highly polar compounds. The choice of mobile phase plays an important role in this column; a total of 1% formic acid significantly improves the appearance of the glyphosate peak and the sensitivity compared to 0.1% formic acid [16]. The main disadvantage of the Hypercarb column is the unpredictability of the loss of retention time of the analyte, which may be related to the contamination of the column [17]. Many authors refer to column problems related to poor reproducibility and column degradation after only a few sample injections. According to the method published by the reference laboratory [11], the Hypercarb column requires long-term preconditioning, which requires at least 50 injections of spinate extract to activate the sites on the stationary phase and achieve satisfactory chromatographic conditions. A multi-residue approach for the extraction of polar analytes is the quick polar pesticides method (QuPPe) developed by the European Reference Laboratory (EURL) for single residue methods (SRMs) [11]. The method is based on acidic methanol extraction. The second method was developed by Herrera-Lόpez et al. and involves extraction with a mixture of water and methanol as an acidic solvent [17,18]. Although the QuPPe method is capable of extracting a wide range of polar analytes, the extract may contain a high concentration of matrix co-extractants, such as lipids in matrices of animal origin, which contaminate the instruments. The only disadvantage of the second method could be the high dilution factor (50- or 100-fold), which leads to a lower sensitivity of the instrument analysis. The determination of glyphosate in environmental samples, including water, has been well researched [6,7,19]. Work has also been published involving glyphosate and its metabolite AMPA in foods of plant origin such as soybeans, corn, grapefruit, oranges, oats and other fruits and vegetables [12,13,17,20,21]. Determinations in complex samples such as foods of animal origin are limited and, to our knowledge, few papers have been published that include all listed glyphosate metabolites [11,14,18,21,22,23,24,25,26,27,28,29]; according to Verdini, few methods have been reported for the detection of glyphosate and its metabolites in foods of animal origin to be used in the multi-year monitoring plan [10].

All the problems mentioned above mean that the analysis of polar pesticides, especially in foods of animal origin, is not very common in control laboratories. Therefore, the aim of this work was to develop a direct LC-MS/MS method for the quantification of glyphosate, AMPA, N-acetyl-AMPA and N-acetyl-glyphosate in foods of animal origin, including adipose tissue, liver, eggs, milk and honey, in order to fill the gap of missing confirmatory methods. The manuscript describes the implementation and validation of a method for the detection of glyphosate and its metabolites in food of animal origin at prescribed levels. Although glyphosate is one of the most commonly used agrochemicals, it is also one of the most difficult products to measure. Mass spectrometry (LC-MS) is currently the most widely used technique for the determination of glyphosate and its metabolites, which is why it was our technique of choice. The aim was to develop a simple and reliable method with good retention stability, short-term preconditioning and a long column life.

## 2. Materials and Methods

### 2.1. Chemicals and Materials

The herbicide standard glyphosate (99.5%) was purchased from Chem Service (Westchester, NY, USA). AMPA (99.7%) was purchased from CPAchem (Bogomilovo, Bulgaria). Glyphosate-13C2, 15N, AMPA-13C, 15N, N-acetyl-AMPA, N-acetyl-glyphosate and N-acetyl-glyphosate-D3 were purchased from Qpp-Lab (Castellana Grotte, Italy). Methanol and formic acid in LC-MS quality were purchased from Sigma-Aldrich (Steinheim, Germany). SPE OASIS HLB (60 mg/3 mL) was purchased from Waters (Milford, MA, USA), while Plexa PAX (60 mg/3 mL) and Plexa PCX (60 mg/3 mL) were purchased from Agilent Technologies (Santa Clara, CA, USA). Ultrafiltration filters (Vivaspin^®^ 5 kDa) with a molecular weight cut-off of 5 kDa suitable for centrifuges were used (Sartorius, Göttingen, Germany). Purified water was used for sample preparation and analysis using a Milli-Q system (Millipore, Merck KgaA, Darmstadt, Germany).

### 2.2. Standard Preparation

Certified pesticide reference materials of known purity from various manufacturers were used for the preparation of standard solutions. If necessary, base solutions were prepared from reference standards in solid form with approximate mass concentrations of 1000 µg mL^−1^ in ψ (water, acetonitrile) = 9:1. Solutions of mixed standards with mass concentrations of 10 or 20 µg mL^−1^ also in the same solvent were prepared from reference standards or pesticide base solutions. The solutions of the working standards were stored for 7 months at +2–+8 °C. Plastic dishes were used for the preparation.

### 2.3. Instrumentation

An Agilent 1290 UHPLC (Agilent Technologies, Santa Clara, CA, USA) and a Hypercarb column (100 × 2.1 mm, i.e., 5 µm particle size) from Thermo Scientific (Waltham, MA, USA) were used for chromatographic analysis. The column temperature was set to 40 °C. Chromatographic analysis was performed using gradient elution, where the mobile phase A was acidified water with 1.2% formic acid and the mobile phase was acetonitrile with 0.5% formic acid in acetonitrile. Elution started at 100% of mobile phase A and decreased linearly to 98% in 4.5 min, then returned in 6 min and continued until 7 min. A re-equilibration step of 3 min was then inserted. The total run time was 10 min. The flow rate was set to 0.35 mL/min and 5 µL of the extracted sample was injected.

During the development of the methods, two other columns with different working modes, the so-called HILIC modes—Torrus Dea and APP (anionic polar pesticide) columns—were tested. The best chromatographic conditions were achieved by using 1.2% formic acid in water (*v*/*v*) and 0.5% formic acid in acetonitrile (*v*/*v*).

Mass spectrometric detection was performed using an Agilent triple quadrupole mass spectrometer (6460C) (Agilent Technologies, Santa Clara, CA, USA). Negative and positive ionisation modes were used to ionise the target compounds. The capillary voltage was set to 2000 V. The drying gas temperature was set at 275 °C, while the sheath gas temperature was maintained at 400 °C. The gas flow was set to 10 mL min^−1^. Data acquisition was performed using MassHunter software version B.07.00.

The quantification of glyphosate, AMPA, N-acetyl-AMPA and N-acetyl-glyphosate were performed using the MRM mode using parameters showed in Table 2. An internal standard (ILIS) was selected for each compound, except for N-acetyl-AMPA for which a deuterated or 13C-labelled IS was unavailable. The monitored transitions for all analytes are shown in Figure S1, Figure S2, Figure S3, Figure S4, Figure S5, Figure S6 and Figure S7.

### 2.4. Sample Preparation

The samples were treated according to the QuPPe method. In brief, the procedure was as follows: a total of 5 g of well-homogenized adipose tissue or 2 g of a liver sample was weighed into a 50 mL centrifuge tube. Then, 10 mL of acidified water (1% formic acid) was added. The tube was then vortexed for 1 min or placed in a rotary shaker for 10 min. In the case of adipose tissue, 5 mL of dichloromethane was added, shaken for 1 min and then centrifuged at 4000 rpm for 15 min. Finally, 1 mL of the aqueous phase was filtered through a syringe filter (0.22 µm) into a plastic vial and was ready for LC-MS/MS analysis. The liver homogenate was centrifuged at 4000 rpm for 15 min at 4 °C. A total of 2 mL of the top layer was transferred to an Eppendorf tube and centrifuged at 14,000 rpm for 15 min at 4 °C. A total of 1 mL of redistilled water was added to the cut-off filters and then 1 mL of the middle-centrifuged layer was added and centrifuged at 6000 rpm for 30–45 min. A total of 1 mL of the centrifuged filtrate was applied to the SPE column, which was previously conditioned with 1 mL of MeOH and 1 mL of 0.5% formic acid in water. A vacuum was applied to collect as much liquid as possible and transferred to a plastic vial for LC-MS/MS. The milk sample (2 g) was weighed into a 50 mL plastic centrifuge cuvette, 10 mL of 1% formic acid (*v*/*v*) was added and shaken by hand or placed in a rotary shaker for 5 min. Then, 2 mL of dichloromethane was added, shaken and centrifuged at 14,000 rpm for 10 min, separating the aqueous and organic layers. The clear upper layer was transferred to a plastic vial and subjected to LC-MS analysis. To a weighed homogenized egg sample (2 g) in a 50 mL plastic centrifuge cuvette, 8.5 mL of 1% formic acid (*v*/*v*) was added and shaken by hand or placed in a rotary shaker for 5 min. Then, it was centrifuged at 4000 rpm for 15 min at 4 °C, allowing the organics to settle. A 2 mL volume of the supernatant was transferred to 2 mL plastic centrifuge tubes and centrifuged at 14,000 rpm for 15 min at 20 °C to precipitate the proteins. A total of 1 mL of redistilled water and 1 mL of the middle-centrifuged layer were added to the ultrafiltration filters and centrifuged at 6000 rpm for 30–45 min. A volume of 1 mL of the centrifuged filtrate was passed over an SPE column (Plexa PCX, 60 mg, 3 mL) preconditioned with 1 mL methanol and 1 mL 0.5% formic acid (*v*/*v*). The eluates were collected in plastic tubes, transferred to plastic bottles and subjected to LC-MS analysis.

To a weighed honey sample (5 g) in a 50 mL plastic centrifuge cuvette, 19 mL of 1% formic acid (*v*/*v*) was added and shaken by hand or placed in a rotary shaker for 5 min. Then, it was centrifuged at 4000 rpm for 15 min at 4 °C, allowing the organic substances to settle. The aqueous layer was filtered through a regenerated cellulose filter (4 mm, 2 µm) into a plastic vial and subjected to LC-MS analysis.

### 2.5. Method Validation Procedure

The methods developed for the determination of residues of glyphosate and its metabolites in food samples of animal origin were validated according to the document SANTE/11312/2021, including the validation parameters of linearity, LOQ, recovery and precision [30].

For the validation, blank matrices from adipose tissue, liver, eggs, milk and honey were used, which do not contain analysed analytes according to the proposed methods.

#### 2.5.1. Linearity

The linearity test was performed by creating a calibration line for all selected ions using matrix-matched standards. Calibration curves were generated by plotting the relative response against concentration (mg/kg) for each analyte. The relative response was calculated as the ratio between the peak area of the natural analyte and the relative ILIS for all analytes except N-acetil-AMPA. Calibration lines were established at a minimum of 3 points by adding a known standard concentration to a pesticide-free matrix prior to the extraction procedure. Solutions of 10 µg mL^−1^ were prepared to create calibration points of 12.5, 20, 25, 40, 50 and 100 ng mL^−1^ for adipose tissue; 5, 10, 20, 50, 80 and 100 ng mL–1 for liver; 5, 8, 10, 20, 50, 80, 100 and 160 ng mL^−1^ for milk and honey; and 2.5, 4, 5, 10, 20, 40, 50 and 60 ng mL^−1^ for eggs by adding known volumes to the sample. For each calibration point, the deviation of the back-calculated concentration (BCC), i.e., the deviation of the concentration calculated by the calibration function (Cmeasured) from the true concentration (Ctrue), was evaluated according to the following expression: (Cmeasured − Ctrue) × 100/Ctrue). According to the document SANTE/11312/2021 [30], the calibration curve was considered linear if for each point, the deviation of the BCC from the true concentration was ≤±20%.

#### 2.5.2. Limits of Quantification

The limits of quantification (LOQs) were determined by adding an appropriate amount of the pesticide to the blank matrix of the sample. The spiking levels established for the LOQs were equal to or lower than the MRLs according to Commission Regulation (EU) No 293/2013 [2]. It was necessary to confirm compliance with the performance criteria, which included recoveries in the range of 70–120% and precision values less than or equal to 20%.

#### 2.5.3. Precision

The precision was determined by adding the standard solution of the analyte mixture to the blank matrix of the sample. The test was performed on at least five measurements for each concentration level, which includes at least 3 different concentration levels. The mean values of the mass concentrations for each spiked concentration level, the standard deviation (SD) and the relative standard deviation (RSD) were calculated. The method is suitable if it fulfils the criteria prescribed in the SANTE/11312/2021 document, according to which the RSD is ≤20%.

#### 2.5.4. Recovery Rate

The recovery rates were calculated for at least 3 concentration levels. At least five measurements were performed for each concentration level. Starting solutions of 10 µg mL^−1^ pesticides were prepared and known volumes were added before starting the extraction to obtain the concentration levels mentioned above. The following validation levels were tested: adipose tissue 0.025, 0.04, 0.05 and 0.1 mg kg^−1^; liver 0.05, 0.1, 0.2, 0.5 and 0.7 mg kg^−1^; eggs 0.04, 0.05, 0.1, 0.2, 0.4, 0.5 and 0.6 mg kg^−1^; milk 0.025, 0.04, 0.05, 0.1, 0.2, 0.4, 0.5 and 0.8 mg kg^−1^ and honey, 0.05, 0.08, 0.1, 0.15 and 0.2 mg kg^−1^.

#### 2.5.5. Measurement Uncertainty

For each analyte, the measurement uncertainty was calculated from the data of the mean of the absolute analytical recoveries and the relative standard deviations covering at least three concentration levels.

The measurement uncertainty was calculated from the data obtained by validation with the coverage factor *k* = 2 (confidence level of approx. 95%) using Expression (1).

(1) $$u(\%)=k\sqrt{{\left(RSD\right)}^{2}+(\frac{100-R}{2\sqrt{3}}}{)}^{2}$$

where *u*(%)—expended measurement uncertainty; *k*—overlapping factor; *RSD*—relative standard deviation (%) and *R*—average recovery (%) from validation.

A default uncertainty value of 50% according to the SANTE criteria [30] should be used to assess compliance with the MRL exceedance legislation. To apply this default parameter, laboratories must demonstrate an experimental *U*′ value of ≤50%.

## 3. Results and Discussion

### 3.1. Chromatography Optimisation

**Figure 2:**
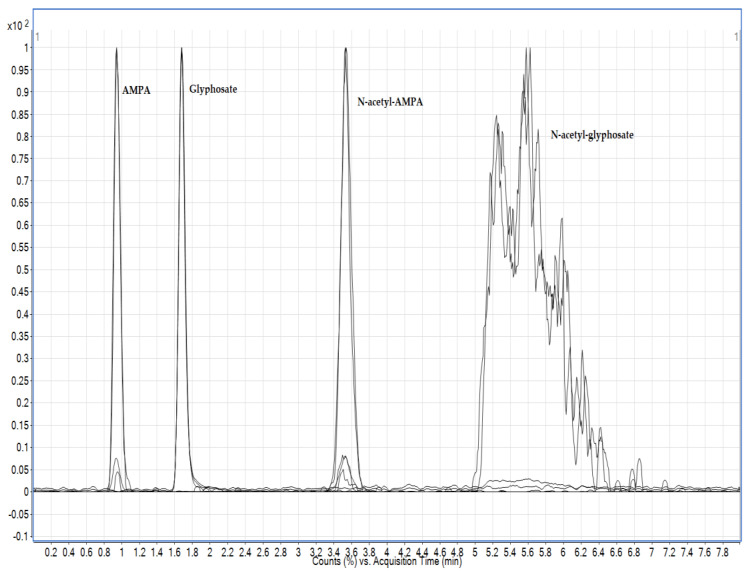
Representative total ion chromatography (TIC) of the standard solution of the analyte mixture in 1% formic acid in water (*v*/*v*), γ = 10 µg mL^−1^ on a Hypercarb column (AMPA, Glyphosate, N-acetyl-AMPA, N-acetyl-glyphosate).

Three different columns were tested in this study: Hypercarb column and two other columns with different modes of action: the HILIC column Torrus DEA (2.1 mm × 100 mm; 1.7 μm, Waters, Milford, MA, USA) and the APP column (anionic polar pesticide column) (5 μm, 2.1 mm × 100 mm). In this study, different mobile phases were tested with water, methanol and acetonitrile with or without the addition of acetic or formic acid. The best chromatographic conditions were achieved using 1.2% formic acid in water (*v*/*v*) and 0.5% formic acid in acetonitrile (*v*/*v*), but the problem arose with the chromatographic separation of N-acetyl-glyphosate, and since it was not possible to achieve chromatographically satisfactory conditions for all the required analytes, the HILIC active columns were discarded and the method was further developed using a Hypercarb column (Figure 2). Formic acid is used in all steps of LC-MS analysis procedures, from sample preparation to chromatographic separation and MS analysis. However, the most important application is its use as an additive in the mobile phase, as even a small amount can improve chromatographic separation and peak shape [31]. Similar to the authors Lee et al. [28], the addition of formic acid significantly increased the sensitivity and improved the appearance of the peak. Although the authors avoid the addition of acetonitrile because of the broadening of the peaks, in this case, the addition of acetonitrile in the mobile phase up to 2% led to an improvement in the appearance of the peaks of N-acetyl-AMPA and N-acetyl-glyphosate. The addition did not affect the stability of the retention time of the analyte or the longevity of the column, as one column lasted up to 2000 injections. Hypercarb columns are known as matrix component-dependent columns. Similar to Nørskov et al. [5], they found that the retention times of the analytes shifted depending on the matrices applied to the column, but the retention times were stable and thus reproducible once the column was saturated with a particular matrix.

Since these are analytes that tend to bind to metals, a slight shift in the mass concentration of the analyte, especially glyphosate, was observed in the solvent analysis performed immediately after the analysis of the standard in the solvent, which may lead to a false positive interpretation of the results. Since all metal lines were replaced by plastic capillaries, the only possible source of binding is the injection unit. The mycotoxin fumonisin has the property of binding to metal surfaces similar to glyphosate; therefore, a similar solution was chosen to reduce the transfer of mass concentration; rinsing the needle in a solution ψ(water, methanol, acetonitrile, isopropanol) = 1:1:1:1, with the addition of 1% formic acid (*v*/*v*) [29].

### 3.2. Optimisation of the Extraction Procedure

In this study, extraction procedures based on the QuPPe approach, the research of Herrera López et al. and the research of Nørskov et al. were tested [5,11,18]. All samples were spiked with standards and ISs prior to extraction. Since glyphosate and its metabolites are very polar analytes, very polar solvents were used: water, methanol and a mixture ψ(methanol, water) = 1:1, each with the addition of 1% formic acid (*v*/*v*). A few researchers achieved the highest recovery rates using 1% formic acid in water [5,8]. Water with the addition of 1% formic acid (*v*/*v*) proved to be the most suitable extraction solvent with the highest detector responses and satisfactory analytical recoveries (>80%). The use of water as an extraction solvent avoids the co-extraction of non-polar compounds and yields a cleaner extract than when methanol is used. Extraction with methanol and a mixture of methanol and water in a ratio of 1:1 (*v*/*v*) resulted in a splitting of the AMPA peak and a lower reaction compared to water extraction. When eggs were analysed according to the method of Herrera López et al. [18], a clear upper layer was obtained in the first analyses after freezing, but when the analyses were repeated, a cloudy layer was formed that was very difficult or impossible to pass through the PCX layer of the sorbent and continued to be cloudy and was therefore not used for further analyses. Due to the strong influence of the matrix, the AMPA analyte was not detected when methanol was used. The final sample was diluted to reduce the amount of matrix injected onto the column to avoid peak splitting, but this resulted in a significant reduction in sensitivity.

For foods of animal origin, especially difficult matrices such as liver and eggs, additional purification steps are required to minimize the presence of interfering factors in the final extract. Adipose tissue consists of large round cells surrounded by connective tissue through which blood vessels run, and in slaughtered animals contains 50 to 90% fat, which is mostly triglycerides. Different volumes of dichloromethane were tested to precipitate the upper fat layer and had no effect on the analytical recoveries of the analytes, as adipose tissue does not contain proteins that would precipitate and entrain the analytes upon addition of dichloromethane, as observed in the study by Nørskov et al. [5] The addition of dichloromethane settles the oil phase and facilitates handling with the water phase of interest and also makes the water phase cleaner in terms of water-soluble lipids. As additional filtrate purification steps, dSPE (C18 and PSA) and SPE (polymeric ion exchange sorbent, PCX) were investigated to remove the influence of the matrix and to achieve the lowest possible limits of quantification. Since the use of extraction methods had no influence on the improvement of the analytical results, no further extraction steps were carried out. Extraction with water avoids the co-extraction of non-polar compounds, resulting in “cleaner” extracts, and no additional purification steps were performed, similar to Chiarello et al. [8].

**Figure 3:**
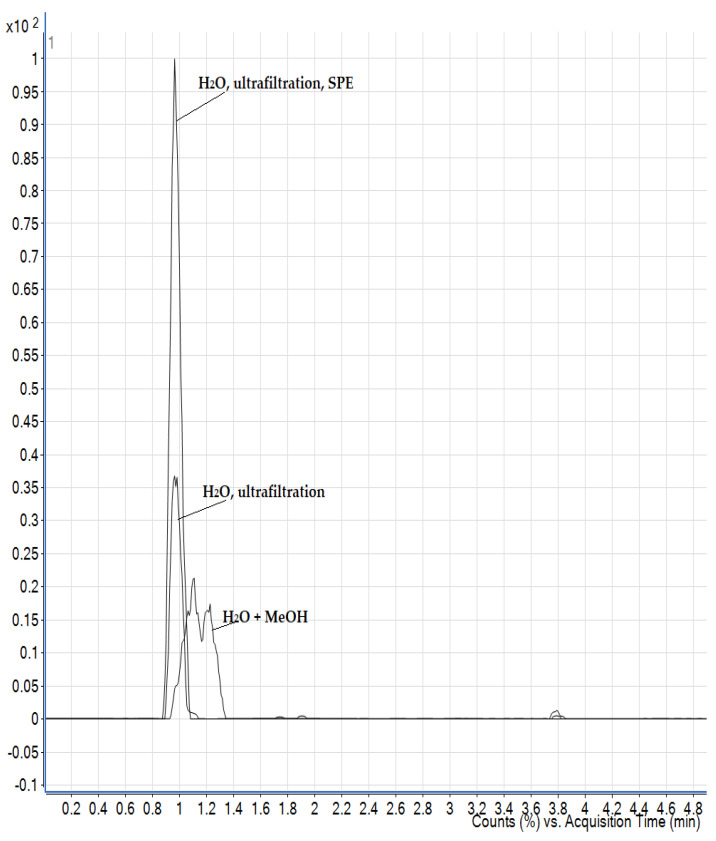
The extracted ion chromatogram for the AMPA analyte in eggs and liver showing increase in the signal when applying both ultrafiltration and solid phase extraction (SPE) compared to only ultrafiltration. The concentration of AMPA in both cases was the same.

The liver is a matrix rich in proteins and phospholipids that can lead to suppression of ions in the MS device, and protein removal is a necessary step in sample preparation. There are several methods for protein removal: acetonitrile, dichloromethane and ultrafiltration with cut-off filters of 10 kDa [5,11,14,18]. Eggs are a particularly problematic matrix because they contain lecithin, an emulsifier that can bind the lipids in the eggs to the extraction solvent, resulting in a cloudy solution. They contain about 75% water, while the rest consists mainly of proteins and lipids. The extracts obtained by extraction with acidified water were additionally purified in various ways, including with dichloromethane, using various SPE sorbents and by ultrafiltration with cut-off filters. Clear filtrates were obtained by ultrafiltration and subjected to LC-MS analysis. For liver and egg samples, the combination of ultrafiltration and PCX resulted in a significantly improved response as matrix interferences were reduced (Figure 3).

**Figure 4:**
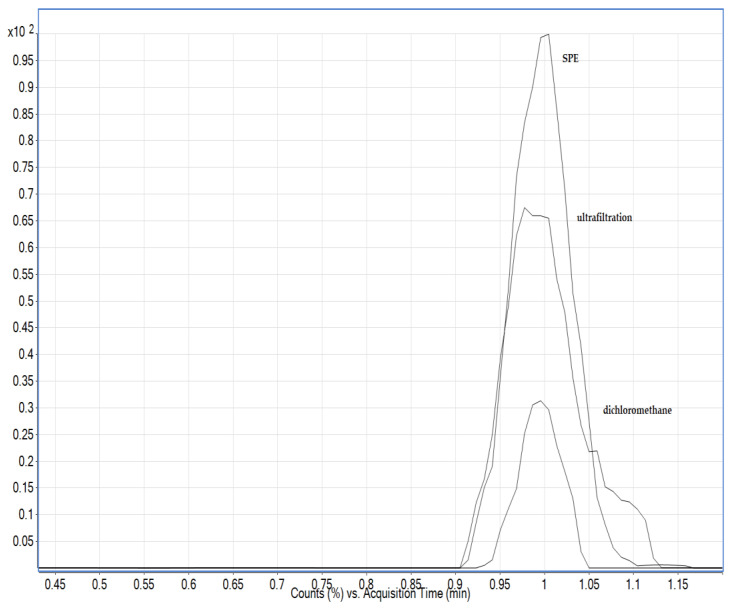
Comparison of the extracted ion chromatogram for the AMPA analyte in milk using different purification steps (SPE, ultrafiltration and dichloromethane). The concentration of AMPA in both cases was the same.

Phospholipids are a major component present in milk and could be extracted along with the analytes. They may accumulate at the head of the analytical column under high aqueous mobile phase conditions and degrade column performance. Therefore, an additional sample clean-up step is required for removing phospholipids and other non-polar compounds, which can be achieved by the addition of dichloromethane or by SPE with PCX sorbent and application of ultrafiltration. However, strong interference was observed in the retention time of the AMPA analyte when using SPE and ultrafiltration (Figure 4); therefore, dichloromethane was used as in the work of Jensen et al. [25]. Milk also contains a high proportion of polyvalent metal cations such as calcium, magnesium and iron ions. These minerals can form chelates with glyphosate, resulting in its loss during protein precipitation process. Extraction solvents containing a Na_2_EDTA-buffered solution can improve recovery. In this work, the effect of the addition of EDTA solution was evaluated together with the other purification steps previously mentioned, and in the case of milk, no improvements were observed; moreover, there was a suppression of the signal.

**Figure 5:**
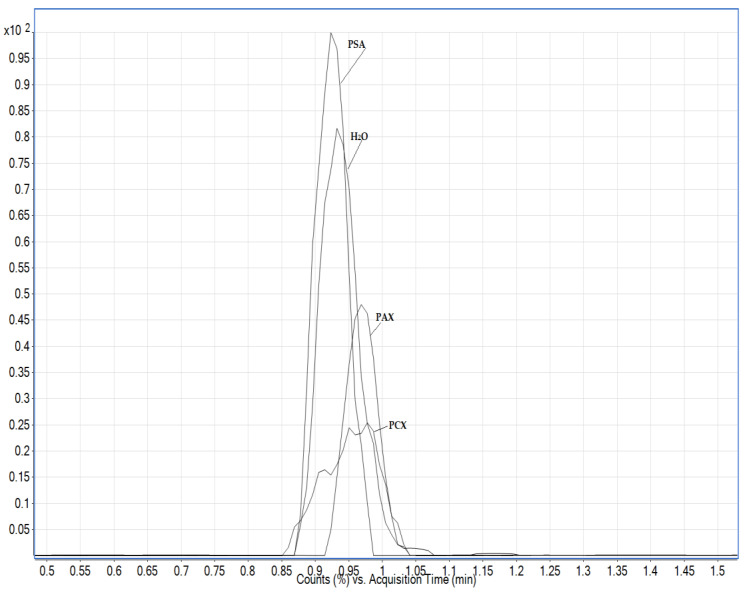
Comparison of the extracted ion chromatogram for the AMPA analyte in honey using different purification steps (PSA, PCX and PAX). The concentration of AMPA in both cases was the same.

Honey as a matrix mostly contains sugars, which are polar compounds and can coelute with glyphosate and its metabolites, especially the AMPA metabolite, and it also contains heavy metals that can interfere with the analytical recovery of the analytes. There is very little in the literature dealing with the analysis of glyphosate and especially its metabolite AMPA in honey, including extraction with acidified water or a modified QuPPE method [11]. Acidified water extraction proved to be the better option as it has less matrix interference and higher analyte response. After extraction with acidified water, purification was performed on the solid phase using two types of polymer sorbents: anionic (PAX) and cationic (PCX). Due to its phosphonate group, glyphosate and its metabolites can be retained and efficiently purified with an anionic sorbent, while sugars as non-ionic compounds are not retained. Dispersive solid phase extraction with PSA sorbent was also used. The use of PCX sorbents leads to strong matrix interference, which affects the retention time of the AMPA analyte. The use of PSA sorbent in dSPE decreases the analytical recovery of glyphosate, while the recovery for the AMPA analyte is highest compared to other purification methods (Figure 5). Since none of the purification steps impaired the achievement of the lower limits of quantification, an additional purification step was omitted.

### 3.3. Analytical Performance

The linearity of the method was tested in the range of mass concentrations of the analytes shown in Table 3. The coefficients of the calibration curves for each analyte in adipose tissue, liver, milk, eggs and honey were determined and are also shown in Table 3. For all analysed analytes, the coefficients of determination were ≥0.95. The linearity study using the back-calculated concentration method gave fully satisfactory results for all analytes.

As part of the validation, the limits of quantification of the analytes in adipose tissue, liver, eggs, milk and honey were determined and presented in Table 4. The limits of quantification were determined as the lowest limit of enrichment of the matrix with analytes at which the criteria of reproducibility (RSD < 20%) and analytical recovery were met (70–120%). The limits of quantification obtained correspond to or are below the currently applicable maximum residue limit (MRL) according to Regulation 293/2013 for glyphosate (Table 1). As the EFSA proposes to consider the MRLs as the sum of the concentrations of glyphosate and its metabolites, the total concentrations would then be numerically higher, but lower limits of determination would have to be achieved distributed among the individual analytes. In this case, the method for adipose tissue and bovine liver would meet the limits of quantification for all analytes. There are very few published papers that cover all the analytes and matrices mentioned above. For example, the authors Herrera López et al. [18] analysed glyphosate and all its metabolites in cow’s milk, pig adipose tissue, kidneys, liver, meat and eggs in their work. The values obtained indicate lower limits of quantification (0.01 mg kg^−1^ for almost all analytes in all matrices) compared to the studies performed, except in the case of glyphosate in liver, where the limits of quantification were 0.2 mg kg^−1^.

Glyphosate and its metabolite AMPA were determined in honey in the work of Chamkasem et al. and quantification limits of 16 ng g^−1^ for glyphosate and 4 ng g^−1^ for AMPA were achieved [22]. By using a coupled system of ion chromatography and mass spectrometry, significantly lower limits of quantification were achieved for glyphosate and AMPA in honey, namely 0.005 and 0.02 mg kg^−1^, respectively [32], while the use of the derivatization step and the online SPE system coupled to the liquid chromatography–mass spectrometry system allowed even lower limits of quantification of 1 µg kg^−1^ for both analytes [33]. Very low limits of quantification for glyphosate and AMPA of 0.0005, i.e., 0.0025 mg kg^−1^ in milk and 0.001, i.e., 0.0025 mg kg^−1^ were obtained in honey, eggs, meat and fish, where the limits were determined by meeting the criterion that the signal-to-noise ratio is greater than 10 [24]. The lowest enrichment concentrations of 0.025 mg kg^−1^ for glyphosate and AMPA were published in milk [14] and limits of quantification of 10 µg L^−1^ [25]. As mentioned above, the EFSA considered in its report a method for monitoring glyphosate, AMPA and N-acetyl glyphosate with a combined limit of quantification of 0.1 mg kg^−1^ and 0.025 mg kg^−1^ for each individual analyte in meat, milk and eggs and 0.2 mg kg^−1^ (or 0.05 mg kg^−1^ for each individual analyte) in liver, kidneys and fat. According to the same report, a confirmatory method for the determination of glyphosate in fat, liver and kidneys and a confirmatory method for the determination of AMPA and N-acetyl glyphosate in all matrices are still lacking and, according to the European Union reference laboratories, there is currently insufficient data to validate methods for the routine implementation of the proposed definition of residues in foods of animal origin.

The accuracy and precision of the method for the determination of glyphosate and its metabolites in adipose tissue, liver, eggs, milk and honey was tested in at least three concentration levels using isotope-labelled internal standards. The results are listed in Table S1, Table S2, Table S3, Table S4 and Table S5. Analytes that meet the criteria of 70 to 120% recovery with an RSD of up to 20% are marked in black, while those that do not are marked in red. Accuracy was calculated as the mean of analytical recoveries of at least five replicates per concentration level. The lowest RSD % values were found for the analyte glyphosate in almost all matrices, the lowest in adipose tissue in the range of 2.7 to 3.9%. The highest RSD % values were obtained for the AMPA analyte in all matrices. Certain analytes did not meet the established criteria at lower concentrations due to lower sensitivity of the quantification or qualification ions or matrix interferences. Satisfactory analytical recoveries for AMPA ranged from 94 to 116%. The lowest analytical recovery for glyphosate was obtained in honey, while the highest recovery of 139% was obtained in eggs, meeting the RSD % requirements. For the analyte N-acetyl-AMPA, the ranges of satisfactory analytical recoveries ranged from 80 to 122%, and the ranges for the analyte N-acetyl-glyphosate were from 85 to 110%. Using isotope-labelled standards, the average values of analytical recoveries in published papers for glyphosate and AMPA in milk were between 84 and 111% with RSD < 8% [14] and between 89 and 107% with RSD < 7.4% [25]. For honey, the analytical recoveries were between 95.2 and 105.3% with an RSD between 1.6 and 7.2% [33] and between 87 and 111% with an RSD < 12% [22].

The data obtained range from 10.8 to 41.9%, as presented in Table 5, which is in line with the criteria prescribed in SANTE/11312/2021, where the standard measurement uncertainty of ± 50% is used for exceeding the MRL when the measurement uncertainty determined by validation is lower. The expected highest values of the expanded uncertainty in almost all matrices were determined for the AMPA analyte. Data available in the literature for the expanded uncertainty of glyphosate and AMPA in honey are 14 and 13%, respectively [32]. The measurement uncertainty for glyphosate was between 15 and 39%. The measurement uncertainty for N-acetyl glyphosate in eggs, fat and milk was between 18 and 48%. Information on the measurement uncertainty for the other metabolites is not available.

## 4. Conclusions

A method was developed for the quantitative determination of glyphosate residues and its metabolites AMPA, N-acetyl-AMPA and N-acetyl-glyphosate using a liquid chromatography–mass spectrometry system in adipose tissue, liver, eggs, milk and honey. The development of the methods addressed the shortcomings identified by the EFSA in its reports, i.e., the lack of a confirmatory method for the determination of glyphosate in fat, liver and kidney and a confirmatory method for the determination of AMPA and N-acetyl-glyphosate in all matrices of animal origin. Good validation parameters according to the SANTE document were achieved for all analytes. As part of the validation of the developed methods, the measurement uncertainty of the analytes was investigated.

For this reason, the method can be considered reliable and sensitive for routine monitoring of polar pesticides. The application of the method in routine analysis will provide data useful for the re-evaluation of risk assessment studies in foods of animal origin. The analysis of glyphosate and its metabolites AMPA, N-acetyl-glyphosate and N-acetyl-AMPA, especially in foods of animal origin, is not very common in control laboratories. Our results provide crucial insights into the analytical efficiency and monitoring of polar pesticides in foods of animal origin, with the aim of achieving an accurate risk assessment of consumer exposure and enhancing the safety of foods of animal origin.

## Figures and Tables

**Table 1:** Maximum residue levels (MRLs) of glyphosate in foods of animal origin according to the current Regulation 293/2013 and proposed MRLs (sum of mass concentrations of glyphosate and its metabolites).

Matrix	Animal Species	Existing MRL (mg kg^−1^)	Proposed MRL According to EFSA (mg kg^−1^)
Muscle	Pig, cattle, sheep, goat, horse, poultry	0.05	0.2
Fat tissue	Pig, cattle, horse, poultry	0.05	0.2
	sheep, goat	0.05	0.3
Liver	Pig	0.05	0.4
	Cattle	0.2	0.7
	Sheep, goat	0.05	0.9
	Horse	0.05	0.7
	Poultry	0.05	0.2
Kidney	Pig	0.5	3
	Cattle	2	7
	Sheep, goat	0.05	10
	Horse	0.05	7
Milk	Sheep, goat, cattle, Horse	0.05	0.1
Eggs	Birds	0.05	0.1
Honey		0.05	No recommendation

**Table 2:** MRM conditions for LC-MS/MS analysis. Transition in underline were used for quantification.

Compound	ISTD	Ion Precursor	Ion Product (*m*/*z*)	Fragment (V)	Collision Energy (V)
AMPA	AMPA-^13^C_2_, ^15^N	110	79 63	70	28 18
Glyphosate	Glyphosate-^13^C_2_, ^15^N	170	88.2 60.2	50	2 12
N-acetyl-glyphosate	N-acetyl-glyphosate-D3	210	124 150	70	12 6
N-acetyl-AMPA	-	152	134 110 63	70	8 8 26
Glyphosate-^13^C_2_, ^15^N	-	173	91 62.2	50	2 12
AMPA-^13^C_2_, ^15^N	-	112	79 63	80	30 16
N-acetyl-glyphosate-D3	-	213	153 126	70	6 12

**Table 3:** Linearity of the method for determination of glyphosate and its metabolites in fat tissue, liver, eggs, milk and honey.

Matrix	Analyte	AMPA	Glyphosate	N-acetyl-AMPA	N-acetyl-glyphosate
Fat tissue	r^2^	0.985	0.996	0.992	0.991
	Linearity/ng mL^−1^	12.5–100	12.5–100	12.5–100	12.5–100
Liver	r^2^	0.967	0.996	0.992	0.993
	Linearity/ng mL^−1^	10–100	5–100	5–100	5–100
Eggs	r^2^	0.978	0.996	0.988	0.987
	Linearity/ng mL^−1^	2.5–60	4–60	4–60	2.5–60
Milk	r^2^	0.984	0.983	0.961	0.986
	Linearity/ng mL^−1^	20–160	5–160	20–160	20–160
Honey	r^2^	0.992	0.993	0.996	0.995
	Linearity/ng mL^−1^	5–160	5–160	5–160	5–160

**Table 4:** Limits of quantifications for glyphosate and its metabolites in fat tissue, liver, eggs, milk and honey.

	Limit of Quantification (mg kg^−1^) in Different Matrixes				
Analyte	Fat Tissue	Liver	Eggs	Milk	Honey
AMPA	0.025	0.2	0.04	0.2	0.1
Glyphosate	0.025	0.05	0.04	0.025	0.05
N-acetyl-AMPA	0.025	0.1	0.04	0.1	0.05
N-acetyl-glyphosate	0.025	0.2	0.04	0.04	0.08

**Table 5:** Expended measurement uncertainty for glyphosate and its metabolites in fat tissue, liver, eggs, milk and honey.

Expended Measurement Uncertainty (%) in Different Matrixes					
Analytes	Fat Tissue	Liver	Eggs	Milk	Honey
AMPA	15.7	41.9	28.6	37.6	32.9
Glyphosate	10.8	13.4	12.4	13.9	31.7
N-acetyl AMPA	13.9	21.0	21.2	20.5	15.7
N-acetyl glyphosate	26.0	12.8	25.1	22.0	22.4

## Data Availability

The original contributions presented in the study are included in the article/Supplementary Materials, further inquiries can be directed to the corresponding author.

## Supplementary Materials

The following supporting information can be downloaded at: https://www.mdpi.com/article/10.3390/foods13152451/s1, Figure S1. Monitored transitions for AMPA-^13^C_2_, ^15^N. Figure S2. Monitored transitions for AMPA. Figure S3. Monitored transitions for glyphosate-^13^C_2_, ^15^N. Figure S4. Monitored transitions for glyphosate. Figure S5. Monitored transitions for N-acetyl-AMPA. Figure S6. Monitored transitions for N-acetyl-glyphosate-D3. Figure S7. Monitored transitions for N-acetyl-glyphosate. Table S1. Precision and accuracy of determination of glyphosate and its metabolite in fat tissue. Table S2. Precision and accuracy of determination of glyphosate and its metabolite in liver. Table S3. Precision and accuracy of determination of glyphosate and its metabolite in eggs Table S4. Precision and accuracy of determination of glyphosate and its metabolite in milk. Table S5. Precision and accuracy of determination of glyphosate and its metabolite in honey.
