# Supplementary material for: Direct Determination of Glyphosate and Its Metabolites in Foods of Animal Origin by Liquid Chromatography–Tandem Mass Spectrometry
Source: Foods. 2024 Aug 2;13(15):2451. doi: 10.3390/foods13152451 (PMC11311992; doi:10.3390/foods13152451)
Supplement: Supplementary file 1 [file foods-13-02451-s001.zip › foods-3123924-supplementary.pdf]

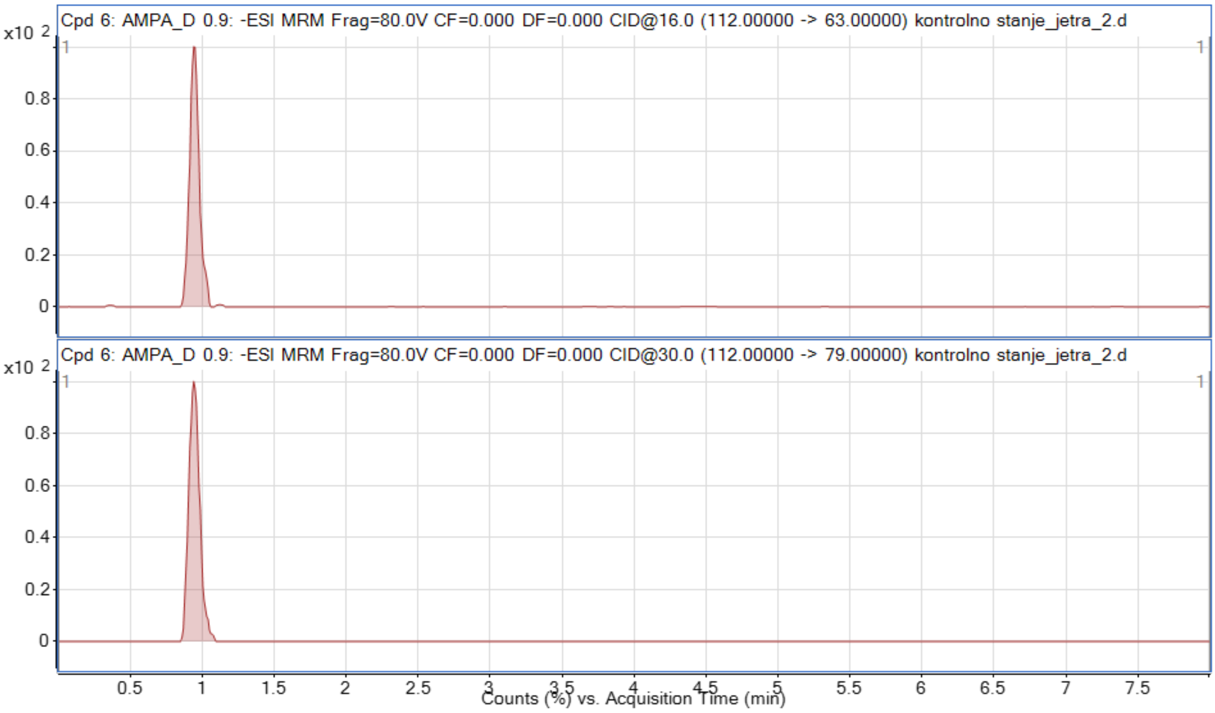

Figure S1. Monitored transitions for AMPA-$^{13}C_2$, ^{15}N.

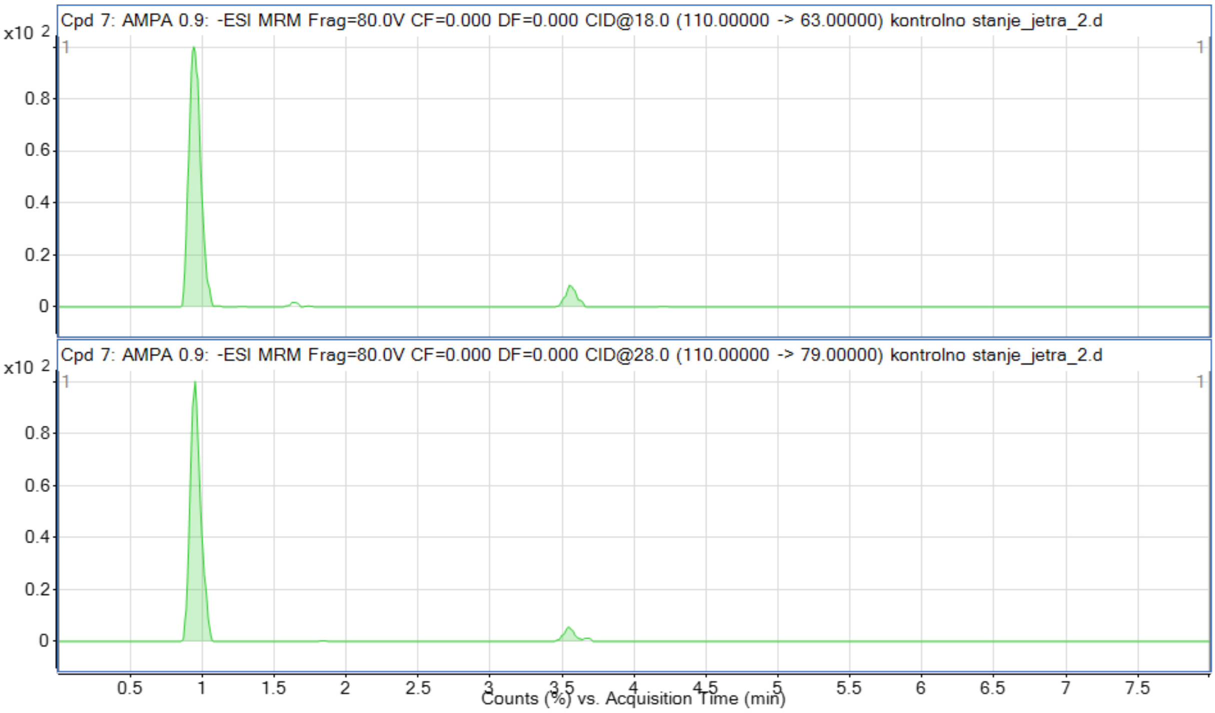

Figure S2. Monitored transitions for AMPA.

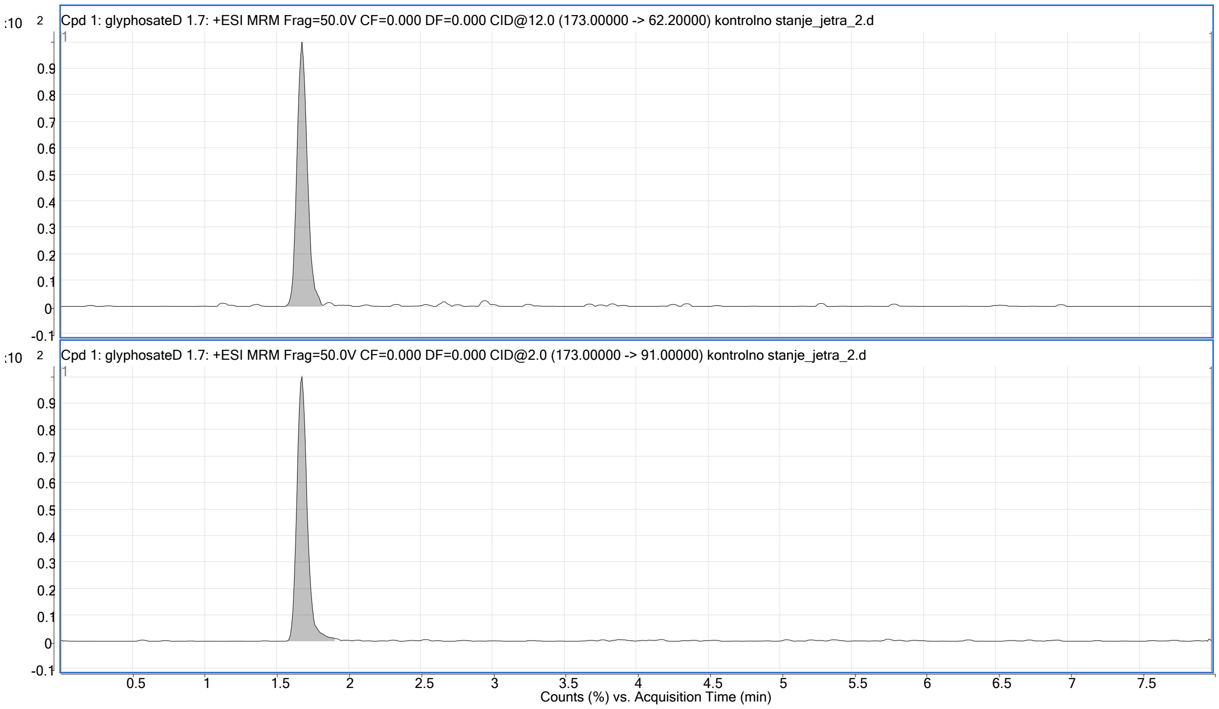

Figure S3. Monitored transitions for glyphosate -$^{13}C_2$, ^{15}N.

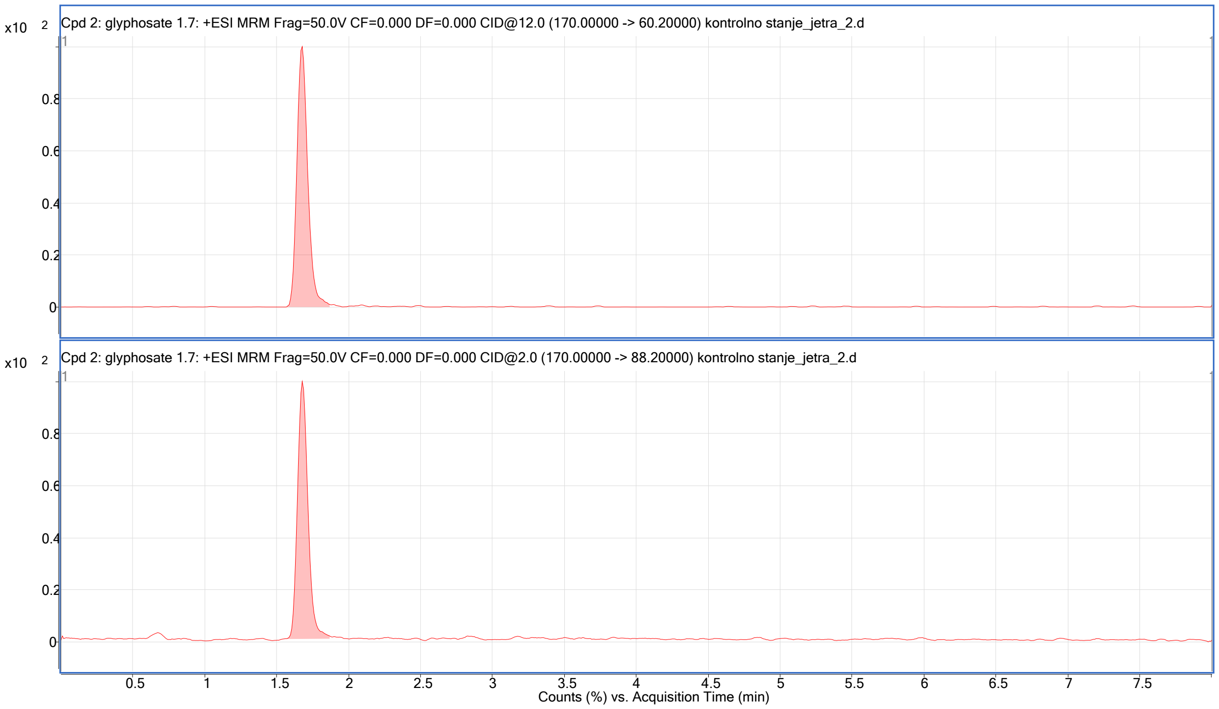

Figure S4. Monitored transitions for glyphosate.

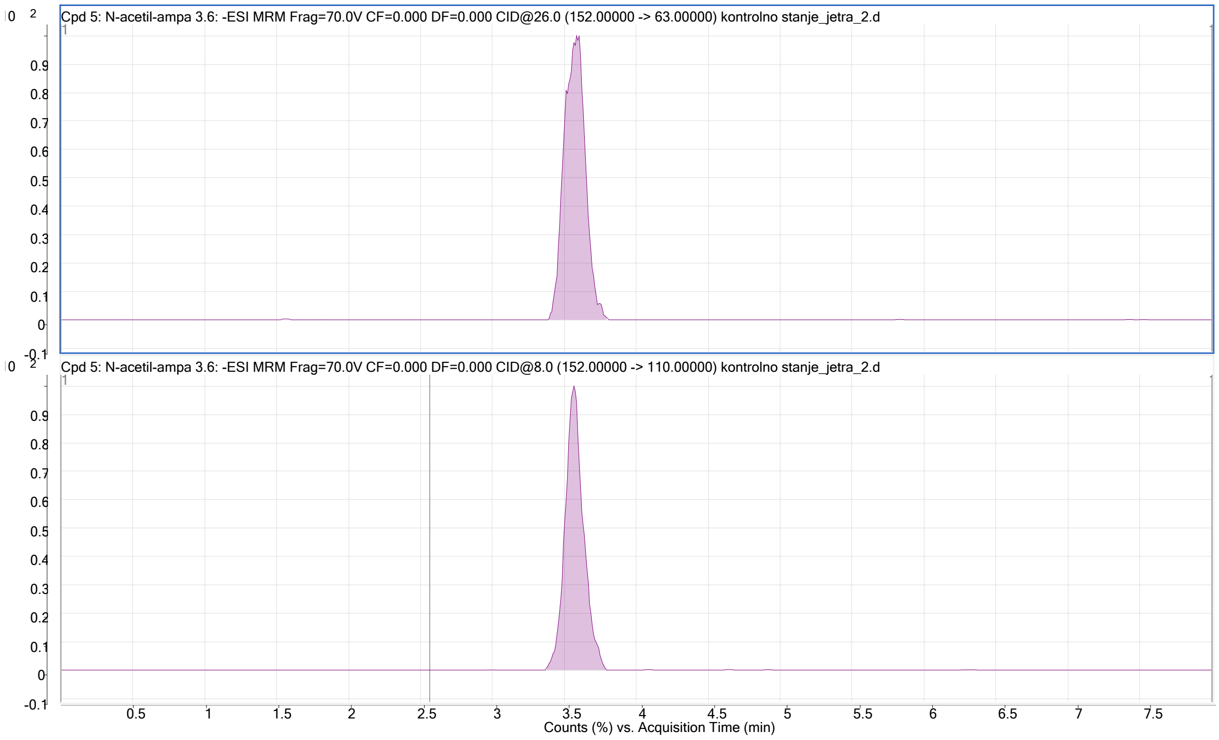

Figure S5. Monitored transitions for N-acetyl-AMPA.

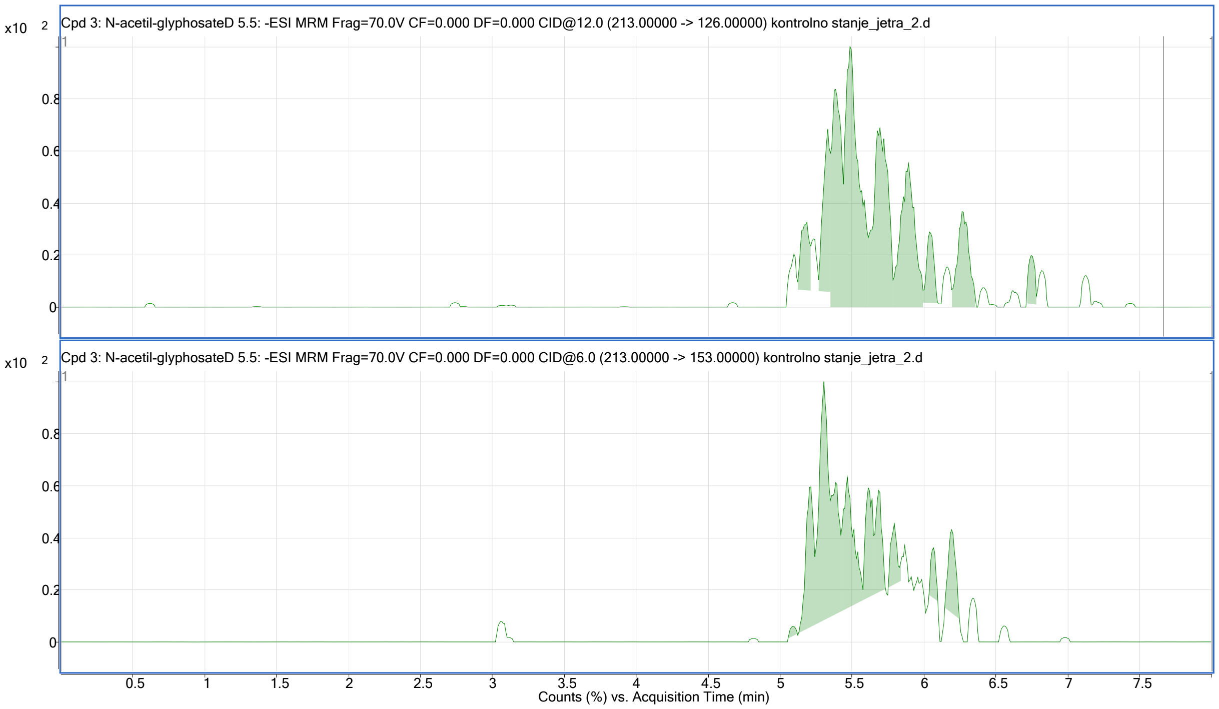

Figure S6. Monitored transitions for N-acetyl-glyphosate-D3.

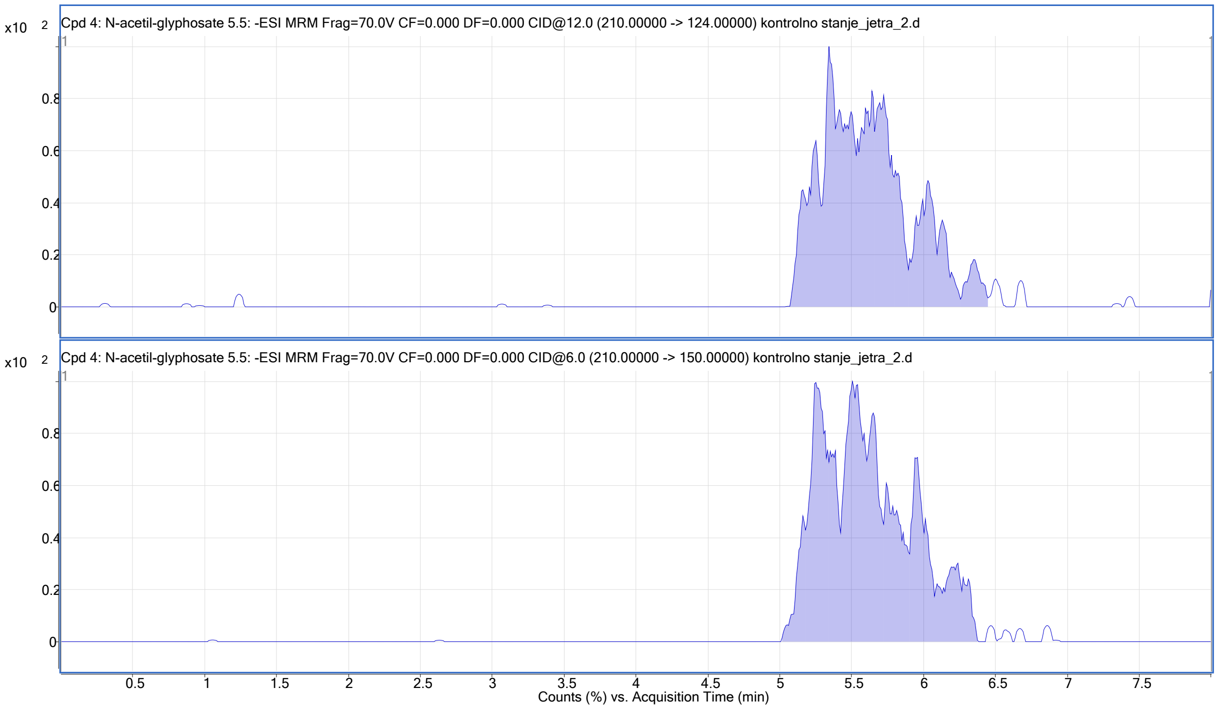

Figure S7. Monitored transitions for N-acetyl-glyphosate.

Table S1. Precision and accuracy of determination of glyphosate and its metabolite in fat tissue.

**Fortification level/ mg kg^{-1}**	**Matrix/ Fat tissue**	**AMPA**	**Glyphosate**	**N-acetyl-AMPA**	**N-acetyl-glyphosate**
	γ/ mg kg^{-1}	0.0262	0.0284	0.0254	0.0263
0.025	R/ %	104.7	113.7	101.7	105.4
	RSD/%	13.2	3.9	13.7	20.8
	γ/ mg kg^{-1}	0.0423	0.0416	0.0409	0.0413
0.04	R/ %	105.7	104.0	102.2	103.2
	RSD/%	11.5	2.97	7.8	17.9
	γ/ mg kg^{-1}	0.0516	0.0502	0.0504	0.0498
0.05	R/ %	103.1	100.3	100.8	99.5
	RSD/%	11.3	2.9	7.5	10.4
	γ/ mg kg^{-1}	0.0982	0.0999	0.1028	0.0996
0.1	R/ %	98.2	99.9	102.8	99.6
	RSD/%	10.9	2.7	6.6	8.4

Table S2. Precision and accuracy of determination of glyphosate and its metabolite in liver.

Fortification level/ mg kg⁻¹	Matrix / Liver	AMPA	Glyphosate	N-acetyl-AMPA	N-acetyl-glyphosate
	γ/ mg kg^{-1}	-	0.06	-	-
0.05	R/ %	-	126.7	-	-
	RSD/%	-	8.7	-	-
	γ/ mg kg^{-1}	-	0.11	0.10	-
0.1	R/ %	-	107.1	99.9	-
	RSD/%	-	6.8	6.7	-
	γ/ mg kg^{-1}	0.23	0.19	0.20	0.22
0.2	R/ %	115.8	97.0	102.0	108.7
	RSD/%	22.4	4.1	5.8	5.7
	γ/ mg kg^{-1}	0.50	0.47	0.52	0.50
0.5	R/ %	100.6	93.9	103.0	101.1
	RSD/%	18.8	4.0	6.9	4.4
	γ/ mg kg^{-1}	0.70	0.69	0.75	0.69
0.7	R/ %	99.6	98.6	106.5	98.9
	RSD/%	20.1	3.11	13.6	7.6

Table S3. Precision and accuracy of determination of glyphosate and its metabolite in eggs.

Fortification level/ mg kg^{-1}	Matrix /eggs	AMPA	glyphosate	N-acetyl-AMPA	N-acetyl-glyphosate
	γ/ mg kg^{-1}	0.025	0.035	0.029	0.025
0.025	R/ %	98.22	139.3	116.0	99.6
	RSD/%	25.3	6.6	12.6	20.6
	γ/ mg kg^{-1}	0.043	0.045	0.041	0.039
0.04	R/ %	106.8	111.6	102.0	98.61
	RSD/%	16.5	3.5	16.2	19.3
	γ/ mg kg^{-1}	0.052	0.053	0.052	0.049
0.05	R/ %	104.6	105.4	104.5	97.13
	RSD/%	12.1	4.4	12.4	16.2
	γ/ mg kg^{-1}	0.10	0.094	0.10	0.095
0.1	R/ %	100.2	94.12	100.5	95.13
	RSD/%	24.1	4.3	9.3	19.4
	γ/ mg kg^{-1}	0.19	0.19	0.19	0.21
0.2	R/ %	98.55	94.18	92.52	104.0
	RSD/%	8.7	5.7	12.6	9.4
	γ/ mg kg^{-1}	0.39	0.39	0.39	0.40
0.4	R/ %	96.83	96.65	97.61	109.9
	RSD/%	3.6	2.7	5,7	4,8
	γ/ mg kg^{-1}	0.51	0.50	0.50	0.48
0.5	R/ %	102.3	99.39	99.77	96.90
	RSD/%	13.1	3.8	7.8	7.3
	γ/ mg kg^{-1}	0.60	0.62	0.61	0.58
0.6	R/ %	98.88	102.6	101.9	97.64
	RSD/%	10.6	1.6	7.4	3.3

Table S4. Precision and accuracy of determination of glyphosate and its metabolite in milk.

Fortification level/ mg kg^{-1}	Matrix / milk	AMPA	Glyphosate	N-acetyl-AMPA	N-acetyl-glyphosate
	γ/ mg kg^{-1}	0.021	0.028	-	-
0.025	R/ %	86.73	113.5	-	-
	RSD/%	73.44	5.63	-	-
	γ/ mg kg^{-1}	0.032	0.041	-	0.034
0.04	R/ %	79.78	101.5	-	85.22
	RSD/%	61.39	18.17	-	17.20
	γ/ mg kg^{-1}	0.038	0.051	-	0.048
0.05	R/ %	76.44	101.5	-	96.42
	RSD/%	54.30	3.58	-	15.72
	γ/ mg kg^{-1}	0.099	0.096	0.12	0.10
0.1	R/ %	92.67	95.63	122.5	100.8
	RSD/%	54.50	3.53	3.57	4.62
	γ/ mg kg^{-1}	0.22	0.19	0.16	0.19
0.2	R/ %	110.5	94.63	80.35	97.63
	RSD/%	17.16	2.07	6.58	10.20
	γ/ mg kg^{-1}	0.38	0.38	0.35	0.37
0.4	R/ %	94.75	95.62	86.90	91.45
	RSD/%	20.46	2.43	12.71	12.38
	γ/ mg kg^{-1}	0.49	0.54	0.59	0.51
0.5	R/ %	97.99	108.6	117.9	103.0
	RSD/%	11.71	14.1	9.14	10.01
	γ/ mg kg^{-1}	0.85	0.78	0.78	0.81
0.8	R/ %	106.5	98.13	97.1	101.1
	RSD/%	19.95	5.63	16.24	3.82

Table S5. Precision and accuracy of determination of glyphosate and its metabolite in honey.

Fortification level/ mg kg^{-1}	Matrix / honey	AMPA	Glyphosate	N-acetyl-AMPA	N-acetyl-glyphosate
	γ/ mg kg^{-1}	0.028	0.018	0.023	0,012
0.02	R/ %	140.2	88.18	114.9	62.08
	RSD/%	25.70	31.84	9.80	27.11
	γ/ mg kg^{-1}	0.041	0.044	0.032	0,041
0.04	R/ %	101.7	110.5	79.05	101,8
	RSD/%	29.83	34.36	13.62	44.71
	γ/ mg kg^{-1}	0.056	0.059	0.054	0.062
0.05	R/ %	112.2	118.1	108.5	123.3
	RSD/%	30.83	20.6	8.90	32.60
	γ/ mg kg^{-1}	0.080	0.085	0.079	0.091
0.08	R/ %	99.6	106.8	99.26	113.3
	RSD/%	34.10	14.07	8.19	14.21
	γ/ mg kg^{-1}	0.10	0.13	0.10	0.099
0.1	R/ %	101.2	86.61	103.7	98.99
	RSD/%	20.95	6.38	10.40	9.33
	γ/ mg kg^{-1}	0.13	0.13	0.15	0.14
0.15	R/ %	87.72	86.61	96.88	92.50
	RSD/%	17.34	6.38	6.35	11.32
	γ/ mg kg^{-1}	0.22	0.21	0.21	0.21
0.2	R/ %	110.0	105.6	103.3	104.10
	RSD/%	10.99	8.00	4.12	9.35
